# Supplementary material for: Are carbon emissions trading and green financial instruments synergistic? -Comprehensive quantitative research based on content analysis
Source: PLoS One. 2024 Mar 7;19(3):e0298601. doi: 10.1371/journal.pone.0298601 (PMC10919601; doi:10.1371/journal.pone.0298601)
Supplement: S1 Data — (ZIP) [file pone.0298601.s001.zip › Data/地级市Z_F工作报告.zip!/╜¡╬≈/╝¬░▓╩╨/└┤╫╘╣1⁄2╓┌║┼í╛┬φ┐╦╩2╛▌═°í┐_2019.docx]

各位代表：

现在，我代表市人民政府向大会报告工作，请予审议，并请各位市政协委员和列席会议的同志提出意见。

一、2018年工作回顾

2018年，是全面贯彻党的十九大精神开局之年，是改革开放40周年。在省委、省政府和市委的坚强领导下，我们深入学习贯彻习近平新时代中国特色社会主义思想和党的十九大精神，从更高层次贯彻落实习近平总书记对江西工作重要要求，践行“创新引领、改革攻坚、开放提升、绿色崛起、担当实干、兴赣富民”工作方针，推进“三个走在前列、两大战略任务”，统筹做好稳增长、促改革、调结构、优生态、惠民生、防风险各项工作，较好完成了市第四届人大三次会议确定的目标任务。

——经济运行保持稳健性增长。主要经济指标增幅保持在全省“第一方阵”，实现生产总值1742亿元、增长8.9%，增幅连续23个季度位居全省前三；规模工业增加值增长9.4%，增幅连续7年位居全省前三；完成财政总收入280.5亿元、增长11.6%，增幅位居全省第二，税收占财政总收入比重突破80%。

——产业结构呈现持续性优化。战略性新兴产业增加值增长20.9%，占规模工业比重提高到19.6%，电子信息首位产业迈入千亿产业行列。单位GDP能耗、水耗分别下降4.7%和5%。服务业增加值占GDP比重提高2.5个百分点，三次产业结构优化为11.9:45.4:42.7。

——创新驱动实现突破性提升。R&D经费支出增长67%，新增高新技术企业111家、达312家，高新技术产业增加值占规模工业比重达到44.1%、高于全省10.3个百分点。井冈山农高区升建工作纳入创新型省份建设重要内容，中科吉安生态环境研究院组建运行，井冈山市入选国家首批创新型县市，泰和工业园区获批省级高新技术产业园区，合力泰成功创建全市首家国家企业技术中心，全市新增院士工作站11家。

——脱贫攻坚取得决定性成效。春季攻势、夏季整改、秋冬会战接续开展，全市实现2.34万贫困人口脱贫、83个贫困村退出，万安县、永新县成功脱贫摘帽，遂川县已接受摘帽评估，万安县“大村长制”经验在全省推广，井冈山市精准脱贫工作入选中国改革开放40年地方改革创新40案例。

一年来，我们主要做了以下工作：

一是统筹兼顾抓重点，经济发展提速增效。始终把加快发展作为重中之重，推动经济速效并进、量质齐升。着力攻项目、稳投资，昌吉赣客专等346个重点项目扎实推进，广吉高速建成通车，井冈山机场二期扩建主体完工，中心城区高铁新区17个项目破难而进，吉安大桥西岸互通工程提前通车，吉安烈士纪念园建成开园；推行工业项目擂台赛、巡回赛“双赛制”，木林森半导体、立讯射频等一批重大工业项目开工竣工；全市固定资产投资增长10.7%，工业技改投资和民间投资分别增长64.2%、15.6%。着力帮企业、强实体，在全省率先成立非公企业维权服务中心，全年累计为企业减负102亿元，新增规上工业企业322家，规模工业利润总额增长14.4%。着力优结构、促升级，开工建设高标准农田27万亩，耕地保护连续6年获省政府表彰，粮食生产实现“十五连丰”，新增六大富民产业种植面积44万亩，稻虾共作综合种养达3.4万亩，“井冈山”区域公用品牌列入省重点建设品牌，全省农业发展大会在吉安成功召开；新增永新三湾、青原东固2个国家4A级旅游景区，万安田北农民画村获批江西省5A级乡村旅游点，吉州窑走进香港、亮相世界，新干金果世界、峡江玉笥养生谷、吉水进士文化园、泰和蜀口生态岛等旅游集聚区加快建设，安福获批江西旅游强县，井冈山旅游公司荣获中国质量奖提名奖，全市累计接待游客总人数和旅游总收入分别增长25.9%、35.1%；光大、兴业银行进驻吉安，新增“新三板”挂牌企业5家，新增数居全省第一，新增贷款310亿元，存贷比提高6.8个百分点，井冈山革命金融博物馆建成开馆；电子商务蓬勃发展，井冈山市、新干县入选全国电商示范百佳县。

二是聚力攻坚破难点，改革开放纵深推进。供给侧结构性改革扎实有效，完成煤炭去产能67万吨、占年度任务的156%，安福龙安等13家煤矿平稳退出并通过省验收。“放管服”改革取得突破，市县两级累计公布依申请类政务服务“最多跑一次”事项7972项，率先在全省推出老年公交卡年审“一次不跑”，企业开办时间压缩至4.5个工作日。开发区改革不断深化，井冈山经开区“一区四园”改革成为全省首创，在全国排名再进11位，获批国家绿色园区。投融资体制和国资国企改革持续深入，市城投资产重组、市旅投混改扩股稳步推进，全市社会融资2100多亿元。开放的广度深度不断拓展，市本级举行电子信息、大健康、现代农业等产业招商活动12场次，成功举办第二届国际存储和计算研讨会、吉厦投洽会；与深圳开展新一轮对接，与中科院、厦门大学、深圳职业技术学院深化人才合作；加强与俄罗斯、阿联酋等“一带一路”沿线国家经贸合作，吉安至莫斯科“井冈山号”中欧班列成功开通，进出口通关时效居全省第一；井冈山机场新增航线3条，通航城市达15个，旅客吞吐量达65.6万人次、居全省支线机场第二位。全市实际利用外资11.7亿美元、增长9.5%，引进内资728亿元、增长10.9%，生产型企业出口18.4亿美元，增长36.4%，增幅居全省第二。

三是打造特色创亮点，环境优势不断巩固。打好污染防治攻坚战，餐饮油烟、燃煤锅炉、秸秆禁烧等十大专项整治扎实开展，全年PM2.5浓度降至40微克/立方米，空气优良天数增幅居全省第一。河（湖、库）长制全面落实，林长制实现全覆盖，赣江中心城区段实行砂石五年禁采，颁布实施全国首部全域水库水质保护地方性条例，全市地表水环境质量指数位居全省第一，新增5个省级生态文明示范县、3个省级绿色低碳示范县，井冈山市获评国家生态文明建设示范市。城乡环境综合整治深入推进，开展彩钢棚整治、渣土密闭清运、“百日净化”等系列攻坚行动，市生活垃圾焚烧发电厂开工建设，所有中心乡镇建成标准化垃圾压缩站。乡村振兴开局良好，高标准完成美丽乡村建设点4065个，农村生活垃圾第三方治理实现全覆盖，高质量通过国家考核验收；绿色殡葬稳步推进，公益性生态公墓辐射到所有村组，全面实行遗体火化。文明城市建设巩固提升，中心城区完成老旧小区 “三改三通三进” 改造22个，惠及居民3万余人，打通“断头路”41条、新改建菜市场10个，评选“模范市民”200名，斑马线“礼让行人”成为吉安靓丽风景。

四是持之以恒聚焦点，百姓福祉持续增进。民生领域财政支出392.5亿元，占财政支出的80.8%。城镇和农村居民人均可支配收入分别为34692元、13820元，分别增长8.6%和10.2%。新增城镇就业5.9万人、转移农村劳动力8.7万人，均超额完成任务。扎实开展城镇贫困群众脱贫解困工作，城乡低保标准分别提高到每人每月580元和340元。适时精准稳控房价，新开工棚户区改造20280套，市中心城区最后一处集中连片棚户区田侯路片区启动改造。国家居家和社区养老服务改革试点扎实推进。“一乡镇一所公办幼儿园”目标全面实现，义务教育学校66人以上“超大班额”基本消除，全域实现义务教育基本均衡。率先在全省实施免费基因检测健康筛查工程，5万名孕妇和新生儿接受基因检测服务，市儿童医院开业运营，吉安荣获首届“健康中国”标志城市。2019年央视春晚吉安井冈山分会场组织有序、精彩惊艳，白鹭洲书院廊桥建成通行，红色采茶歌舞剧《杜鹃花开的地方》在省内外巡演；永丰恩江考古发现全省规模最大、保存最好的古县城遗址，永丰县获评省级历史文化名城，永新县获评中国民间艺术之乡；吉安城市绿道马拉松赛和第四届老年人运动会成功举办。平安吉安、法治吉安建设深入推进，信访维稳、安全生产、食品药品监管工作有效加强，扫黑除恶专项斗争取得阶段性成果，打掉黑社会性质组织9个、恶势力犯罪团伙85个，查处涉黑涉恶腐败“保护伞”案件26起，社会大局保持和谐稳定。

五是“刀刃向内”通堵点，政府建设不断加强。“五型”政府建设深入开展，摸排出171个制约高质量跨越式发展的瓶颈问题，并制定了解决方案；大力整治“怕、慢、假、庸、散”作风顽疾，倡导无事不扰、有求必应的“店小二”精神。法治政府建设扎实推进，完善行政决策合法性审查和政府法律顾问制度，《吉安市烟花爆竹燃放管理条例》颁布实施，认真办理人大代表建议和政协提案。坚决全面彻底肃清苏荣案余毒，查处了一批侵害群众利益的不正之风和腐败问题，严格落实中央八项规定及实施细则精神，开展民生资金、领导干部经济责任审计，“三公”经费下降1.3%，廉洁政府建设进一步加强。

切实抓好国防教育、拥军优属和退役军人事务工作，人民防空、民族宗教、外事侨务、对台、档案、地方志、防震减灾、气象水文、供销、红十字会、残联等各项工作取得新成效。

各位代表，大海不拒细流，故能成其大；泰山不却微尘，故能成其高。2018年，我们充实而坚定地一步一步走来，收获了一点一滴的成绩。这些成绩，犹如涓涓细流汇聚成全面小康的洪流，犹如点点星光照亮全面小康的征程。成绩来之不易，这是以习近平同志为核心的党中央关心关怀的结果，是习近平新时代中国特色社会主义思想特别是习近平总书记对江西工作重要要求科学指引的结果，是市委带领全市人民拼搏奋斗的结果。在此，我代表市人民政府，向全市人民，向各民主党派、人民团体和社会各界人士，向驻吉人民解放军、武警官兵，向中央和省驻吉单位，向所有关心支持吉安发展的同志们、朋友们，致以衷心的感谢和崇高的敬意！

一年来的砥砺奋进，让我们深刻体会到，思想引领是最根本的引领，我们坚持以习近平总书记视察江西、吉安时重要指示为统领，确保各项工作沿着正确方向破浪前行；作风优势是最强的优势，我们大力践行“担当实干、马上就办”作风，把井冈山精神演绎到各个领域、各个岗位，激发出推动发展的强大动力；人心是最大的政治，我们站稳群众立场，以造福人民为最大政绩，赢得了群众的大力支持。

需要向代表说明的是，受国内外市场需求增速放缓和社会心理预期等因素影响，去年全市固定资产投资、社会消费品零售总额、外贸出口等指标增长低于预期目标。同时，我们清醒看到，工作中还有不少问题和不足，主要是：经济总量不大、人均值偏低，产业层次不高、创新能力不足，实体经济仍有不少困难；公共服务领域还有不少短板；生态环境治理压力较大，推进和巩固脱贫攻坚任务艰巨；少数干部思想保守、观念落后、作风不实、缺乏担当，个别部门推诿扯皮、敷衍塞责，慢作为、不作为、假作为，等等。对这些问题，我们一定高度重视，采取更加坚决有力的措施加以解决。

二、2019年工作安排

今年是新中国成立70周年，是决胜全面建成小康社会、实现第一个百年目标的关键之年。当前，世界面临百年未有之大变局，中美经贸摩擦仍有不确定性，国内经济下行压力加大、结构调整阵痛凸显，对我市发展带来挑战。但我市发展稳中有进的基本态势没有变，“四化”协同发展、并联发展的阶段性特征没有变，人文生态优势日益强化，交通区位优势日益凸显，今年将正式迎来“高铁时代”。综合分析，吉安正处在创新驱动、变道超车、后发赶超的重要战略机遇期。机不可失，时不再来，历史的机遇正等待坚定者、奋进者、搏击者。我们要在抢抓机遇中创造未来，书写吉安发展新篇章。

今年政府工作的总体要求是：以习近平新时代中国特色社会主义思想为指导，深入贯彻落实党的十九大、十九届二中、三中全会、中央经济工作会议以及省委十四届六次、七次全会和市委四届七次全会精神，从更高层次贯彻落实习近平总书记对江西工作的重要要求，坚持稳中求进工作总基调，坚持新发展理念，坚持高质量跨越式发展首要战略，以深化供给侧结构性改革为主线，强化创新驱动，狠抓改革攻坚，深化开放合作，着力打好“三大攻坚战”，着力建设现代化经济体系，着力推动区域协调发展，着力加快绿色崛起步伐，着力保障和改善民生，着力推进“五型”政府建设，确保经济平稳健康发展和社会和谐稳定，为实现全面小康、加快绿色崛起打下决定性基础，以优异成绩庆祝中华人民共和国成立70周年。

今年发展的主要预期目标是：生产总值增长8.8%左右，财政总收入增长7%，一般公共预算收入增长6%，规模工业增加值增长8.8%左右，固定资产投资增长9%，社会消费品零售总额增长11%，实际利用外资增长6%；城镇、农村居民人均可支配收入分别增长8%左右和9.5%左右，居民消费价格指数上涨3%以内，城镇登记失业率控制在4.5%以内，节能减排完成省下达任务。

各位代表，吉安自古享有“江南望郡”“金庐陵”的美誉；奋进新时代，我们要共绘江南望郡金庐陵新的美好画卷，这是全市人民的期盼，我们要敢担当、勇拼搏，以力举千钧的拼劲、力拔头筹的干劲，干出一个新吉安。工作中，我们将全面贯彻中央“六稳”部署，做到“四个坚定不移”：一是坚定不移在高质量发展的康庄大道上奋力赶超。坚持质量第一、效益优先，强化创新引领，培育新动能，推动变道超车、换车超车。二是坚定不移在改革开放的时代浪潮中楫水行舟。坚持思想解放为先导，把改革贯穿全过程，以更大力度挺进开放前沿，融入全球产业链、价值链、供应链。三是坚定不移在绿色崛起的科学路径上稳步前行。大力践行“绿水青山就是金山银山”理念，锻造绿色崛起这根“金扁担”，一头挑起金山银山，一头挑起绿水青山。四是坚定不移在全面小康的伟大征程中奋力冲刺。坚持培优补短，尤其要打好防范化解重大风险、精准脱贫和污染防治三大攻坚战，咬定目标、只争朝夕。

围绕上述目标和要求，重点抓好以下九个方面的工作：

（一）立足特色优势，扩增量、优存量，加快构建现代化产业体系。坚持向特色优势要竞争力，集聚集群、去旧育新，推进产业迈向中高端。

聚焦推动电子信息首位产业扩量提质。实施三年倍增行动，开展产业评估，编制实施技术路线、应用领域、区域分布路径图，年内主营业务收入达1300亿元，占据全省京九电子信息产业带首要地位，打造全国重要电子信息产业基地。突出全域化布局，以吉泰走廊为龙头，各县市区差异化布局细分产业，年内井冈山经开区、吉安县均突破300亿元，吉州区迈上200亿元台阶。突出全链式发展，对接中国航天十二院“星融网”应用，举办集成电路论坛，壮大通讯传输、触控显示、LED照明等百亿集群。突出全要素保障，落实“特惠八条”，在合力泰百亿企业基础上，推动木林森、立讯跨入百亿行列。

持续推动主导产业做优做强。壮大生物医药大健康产业，新增道地中药材种植面积5万亩以上，推动种、医、游、养多业融合，力争大健康产业主营业务收入突破700亿元，加快把这一利国利民的幸福产业打造成吉安第二个千亿产业。推进“互联网+”“大数据+”“智能+”，发展工业设计、检验检测等服务型制造，加快企业智能化改造，培育工业互联网平台和云上企业，对接“03专项”成果，谋划布局5G应用，发展数字经济，打造全省先进制造业示范区。发展新型材料、绿色食品产业，加快永丰碳酸钙科创城建设，培育遂川硅基材料等重点集群，完善“1+4”产业体系。

乘势推动新兴产业扩量成势。深化“全景吉安、全域旅游”战略，推进15个集聚区全面成型、串点成线，力争吉州窑景区纳入国家5A级景区质量评审名录，全市新增1-2个国家4A级景区，壮大乡村旅游、文化旅游、高铁旅游新业态，全面形成“三区一网”优质旅游发展格局。大力发展通用航空、军民融合产业，加快省级军民融合创新示范区建设，完成井冈山机场二期扩建，新增东北方向2条经停航线，开工建设桐坪机场二期、武功山通用机场，支持吉安航校、桐坪航空产业园建设。

坚定推动传统产业改造升级。实施工业技改重点项目100个以上，打造数字车间、智能工厂、绿色企业，确保工业技改投资增长30%以上。重拳整治“散乱污”企业，确保“地条钢”零存在。推进食品纺织、建材化工等产业工艺升级和设备改造，抓好新干县盐卤药化省级传统产业优化升级试点。

（二）推动双侧发力，强实体、扩需求，筑牢高质量跨越式发展根基。坚持以供促需、以需优供，做强实体经济，稳定市场预期，稳住有效内需，推动经济提质增效。

壮大民营经济。坚定贯彻“两个毫不动摇”方针，全面落实减税降费政策，阶段性下调养老保险缴费比例，一般工商业平均电价再降低10%，加快涉企行政事业性收费“清零”，年内再为企业减负100亿元以上。实施“转企升规”工程，新增个升企450家以上、规上企业200家以上、省级“小巨人”企业2家、省级“专精特新”中小企业15家以上。发挥非公企业维权服务中心作用，规范涉企案件办理，保护企业家合法权益。弘扬企业家精神，实施企业家成长计划，继续办好“领航井冈”企业家高级研修班，加大优强企业和优秀企业家表彰力度，推动民营经济再立时代新功。

激活金融活水。加快创建国家普惠金融改革试验区，筹建普丰农业保险公司。扩大无还本续贷、信用贷款、知识产权质押贷款适用范围，提高“财园信贷通”“财政惠农信贷通”精准度，做大转贷基金，完善一站式金融服务平台和担保体系，促进银税征信互认、银企信息互通，着力解决小微企业融资难、融资贵问题，存贷比再提高2个百分点，引导更多金融活水浇灌实体经济。推进企业上市“映山红”行动，跟进科创板注册制政策，建立30家左右重点企业上市后备库，力争安福海能实业创业板上市、青原区明辉光电境外上市。

稳定有效投资。开展“大干项目年”活动，围绕重点领域和薄弱环节，安排401个重点项目，年度完成投资1240亿元。年内完成昌吉赣客专、蒙华铁路、新干航电枢纽、赣江三级航道建设，加快推进井冈山航电枢纽、国省道改造等项目，推动宜井遂高速、大广高速吉安至南康段扩容改造、长赣铁路、衡茶吉铁路吉井段动车改造尽快开工，做好吉抚武温铁路前期工作。加大产业项目建设，推进集群式项目满园扩园行动，设立重大工业项目投资引导资金，确保国家级、省级开发区年内分别至少引进1个投资过50亿元、20亿元产业项目。强化融资与服务保障，加大政策性银行信贷、地方政府专项债券争取力度，积极发行绿色债、管廊债等专项债，引进第三方图审中心，完善项目代办服务制，推行企业投资项目审批承诺制，上半年要实现政府、企业投资项目审批时间分别压减至79个和60个工作日以内，让企业多用时间跑市场、少费功夫跑审批。

挖掘消费潜力。实施商贸消费升级三年攻坚行动，优供给、促消费，培育家政养老、文体教育等消费热点，扩大共享经济、远程定制、婴幼儿照护等新兴消费。优模式、促消费，加大商业综合体、特色商业街区建设，抓好国家电子商务进农村综合示范县建设，实施吉安产品“两上三进”和“网上行”推广工程。优环境、促消费，落实个税专项附加扣除政策，推进内外销产品“同线同标同质”工程，加快质量强市建设，强化顾客公民心态，严厉打击假冒伪劣行为，让百姓放心消费、乐享消费。

防范化解重大风险。强化忧患意识和底线思维，坚决打好防范化解重大风险攻坚战。防控金融风险，严厉打击非法集资、恶意逃废债行为。严守政府性债务红线，严禁新增隐性债务。健全房地产市场健康发展长效机制，完善土地出让公开公示制度，稳地价、稳房价、稳预期。加强网络舆情监控，严厉打击网络诈骗等犯罪行为。健全重大决策风险评估和防控机制，坚持在推动高质量发展中防范化解风险，坚决守住不发生系统性区域性风险的底线。

（三）强化创新引领，优平台、聚人才，增强经济创新力和竞争力。实施创新型城市建设三年行动，以科技创新为核心，加快打造创新驱动强引擎。

完善产学研用金一体化科技创新体系。加大财政投入，调动企业、院校和工程业主等各方面研发积极性，力争全市R&D经费支出占GDP比重达到1.4%。强化企业主体作用，落实企业研发准备金和首台（套）政策，推行“科贷通”，年内培育“瞪羚”企业10家左右，新增高新技术企业100家，力争全市规上工业企业研发投入强度达到0.8%。组织实施协同创新重大专项，促进创新链、产业链、资金链、政策链“四链融合”。

强化平台与人才支撑。加快井冈山国家级农高区升建步伐，抓紧规划编制和基础设施建设，年内力争引进省级以上科研院所10家，打造南方红壤丘陵产业融合示范区；推进吉安高新区融入鄱阳湖国家自主创新示范区建设，推动安福、永丰、吉水工业园创建省级高新技术产业园，促进中科吉安生态环境研究院成果转化，开展创新型县市区创建。推进“庐陵英才”计划，既通过柔性引才等方式，引进高层次人才，又以企业为主体引进实用型专技人才，培养庐陵工匠；加大留才力度，力争高校职院毕业生留吉率达60%以上；加强人才服务，新建人才公寓2000套，让人才有事业、家属有就业、孩子有学业。

激发“双创”活力。设立创新创业引导基金，鼓励发行双创金融债券，降低创业担保贷款反担保门槛，健全创业资金连续帮扶机制。加强创业孵化基地建设，推动众创空间园区全覆盖，年内新增省级以上创业孵化平台2个，实现省级创新平台县域全覆盖，推动“大众创业、万众创新”竞相迸发。

（四）深化改革开放，破难题、拓空间，持续释放发展的动力活力。强化目标导向和问题导向，以处理好政府与市场的关系为核心，推动改革再出发、开放再提升。

深化“放管服”改革。拓展“最多跑一次”改革，年内依申请类政务服务事项“一次不跑”“只跑一次”办理率不低于80%。推进“一门式”服务、“一窗式”办理，原则上年内依申请类政务服务事项全部进驻服务中心，实行延时、错时、预约服务，“一窗式”受理率达70%以上。推进“一网通办”，6月底前建成一体化在线政务服务平台，并与全省平台对接，依申请类政务服务事项网上可办率不低于70%。全面推开“证照分离”改革，实行“多证合一、一照一码”，实现企业登记全程电子化。推进“互联网+监管”系统建设，全面实施“双随机、一公开”市场监管，对违法者严惩不贷、对守法者无事不扰。

深化开发区改革创新。深入推进三年攻坚行动，促进开发区优化整合，年内开发区主营业务收入突破3000亿元。开展井冈山经开区“一区四园”改革效果评价，加快青原产业园提质增效和富滩产业园扩区调规；加快经开区西区改造升级，启动小微企业产业园建设，推动低效企业“腾院上楼”、僵尸企业 “腾笼换鸟”；完成金鸡湖创新小镇人才公寓、创新大厦主体建设。加快吉安高新区凤凰园区“北拓西扩”，启动娑罗山隧道工程前期工作，推进智能制造产业园和创新创业产业园建设，吉安高新区年内主营业务收入突破500亿。实施“两型三化”园区管理提标提档行动，年内所有开发区智慧平台与区内规上企业互联互通，实现“园区事、园区办结”；树立“亩均论英雄”导向，加快消化批而未用土地，新建多层标准厂房300万平方米以上，推进“新九通一平”建设，提升园区产城融合水平。

推进重点领域改革。全面完成政府机构改革，加快职权整合，同步完善清单、优化流程，实现由“物理集中”向“化学反应”的转变。加大国资国企和投融资体制改革，推动投融资平台优化重组、市场化转型，组建以市城投和新庐陵为龙头的集团公司，实现“规模千亿级、信用上等级”。深化财税体制改革，全面实施预算绩效管理，优化财政支出结构，提高财政资金配置效率。统筹推进行政执法、事业单位、社会民生等各项改革。

推进开放提升、区域协同。全面融入“一带一路”建设，加快井冈山出口加工区升级综合保税区，发挥中欧班列示范效应，布局“海外仓”，推动吉安产品“走出去”，力争生产型企业出口突破20亿美元。全面融入长江经济带、京津冀协同发展战略，深度对接长三角一体化、粤港澳大湾区、海西经济区、成渝经济区，形成“南下北上”“东进西出”开放格局，打造产业梯度转移承接示范地和双向开放新高地；聚焦招大引强，开展 “三请三回” “三企”入吉，走出去请、上门去引，吸引更多国内外客商到吉安投资兴业。全面融入全省区域发展战略，对接新一轮苏区振兴政策，实施“主轴驱动、区域协同”战略，推动高铁新区、井冈山经开区、吉安高新区、井冈山农高区互动发展，促进吉泰城镇群融合发展，推进新宜吉转型合作，将吉安打造成北连大南昌都市圈、南接赣州省域副中心的中部脊梁。

（五）提升城市内涵，强功能、提品质，建设秀美便捷有序宜居宜业宜游之城。实施城市功能与品质提升三年行动，抓好“八大行动、百项工作”，让城市更加精致精美、宜居宜业。

推动高铁新区加快成型。推进“1+5”高铁新区建设，打造产城融合城市新区和经济增长新板块。中心城区高铁新区按照“两个龙头”定位，探索土地出让、工程招标、经营招租、产权交易“四位一体”模式，完成阳明西路、君华大道、站前大道等“三纵五横”路网建设，启动“五指峰、三中心”、3个安置小区、东塘大道地下综合管廊等项目建设。支持配合高铁建成开通，统筹沿线5个县高铁新区产业布局和开发建设，突出电子信息、休闲旅游、住宅商贸业态布局，深化沿线环境整治和通道绿化美化，真正让高铁开通之时成为展示美丽吉安之机。

推进老旧城区更新改造。深化老旧小区“三改三通三进”三年行动，吉州区、青原区分别再打造10个以上精品示范点，推进住宅建筑加装电梯试点，全市完成30%以上的背街小巷改造任务。加大棚户区、城中村改造力度，新开工改造棚户区3.8万套。加快城市“双修”和海绵城市建设，实施纳管截污、雨污分流、引水入城工程，全面消除城区黑臭水体，开工建设中心城区应急水源项目。

持续完善城市功能配套。着力强功能，中心城区新建19个停车场，新建改建25个标准化菜市场，新增停车位4000个，完成公交站台改造和4个充电桩场站建设，推进亚行贷款高品质公交提升工程。着力保畅通，中心城区年内全面打通“断头路”，加快105国道东改、赣江大桥、桐坪机场快速通道建设，建成神冈山大桥，实现“内成网、外成环”。着力提品质，加强文化街区保护，推进夜景亮化工程，完成中心城区滨江生态公园、神冈山公园、白雾山公园建设，全市建成区绿地率提高到41%以上。着力强管理，落实《吉安市市容和环境卫生管理条例》，深化违章搭建、防盗网、小区管线、批而未建围挡地块专项整治，推动出台《吉安市住宅物业管理条例》，中心城区试行“以克论净”环卫管理模式，道路机扫率达到85%以上，不断提高城市管理精细化水平。加快智慧城市建设，巩固全国文明城市创建成果，年内建成市县一体政务云和大数据中心，推进井冈山市、新干县创建全国文明城市，抓好吉水八都等市级以上特色小镇建设，不断创造韵味别致、情景交融的城市生活。

（六）推进乡村振兴，强筋骨、铸魂魄，加快实现农业强、农村美、农民富。坚持农业农村优先发展，推动资金、人才向“三农”倾斜，促进农业农村现代化。

强健绿色有机的产业筋骨。稳定粮食生产，推进粮食收储“并库升级”，新开工建设高标准农田32.5万亩，确保粮食总产稳定在80亿斤以上。融入全省农业结构调整“九大工程”，新增六大富民产业种植面积49.5万亩、稻虾综合种养3万亩，建成运营30个生猪生态循环养殖小区，扩大“井冈山”区域公用品牌影响。推进一二三产融合发展，加大农产品精深加工和包装营销力度，完善冷链物流、农产品质量追溯等体系，培育新型经营主体，创建省级田园综合体；引入专业公司盘活闲置农房，发展精品民宿、休闲农业，推动乡村主要由卖产品向卖风景、卖文化、卖体验转变。健全农机农技、气象水文、供销合作等社会化服务体系，推进中型灌区续建配套节水改造，不断提高农业综合生产能力。

塑造美丽宜居的环境形体。推动美丽乡村建设提质扩面，抓好井冈山、吉水、安福省级美丽宜居试点县建设，争创一批省级示范村点。加快补齐农村基础设施短板，推进农网改造、光纤下乡，巩固农村饮水安全，新建农村公路720公里，基本实现农村公路组组通，完善“8+4”公共服务设施。深化农村人居环境整治，学习浙江“千万工程”经验，推进农村污水处理设施建设，实施城乡“厕所革命”三年攻坚，强化农村建房管控，开展村庄“净化行动”，动员群众清除灰尘、收拾杂物，整理庭院、修饰房屋，彰显新村新貌。

铸造富裕文明的新风魂魄。深化移风易俗行动，推进绿色殡葬改革，完善公墓等殡葬服务设施，抓好“三沿六区”散埋乱葬治理。发挥红白理事会、村规民约和新乡贤作用，推进农村“法律明白人”培养工程，促进自治、德治、法治互动。传承优秀乡土文化，加大古村落、农耕古迹的保护与修复力度。推进农村承包地“三权分置”和集体产权“三变”，因地制宜发展资源开发型、资产经营型、休闲观光型村集体经济，年内80%以上的村经营性收入突破5万元。推进新型职业农民培育工程和“一村一名大学生”工程，为乡村振兴注入强劲动力。

（七）加强生态建养，控源头、抓治理，打造最具普惠的生态福祉。保持生态文明建设战略定力，积极融入国家生态文明试验区建设，以铁腕之力整治环境污染，以大爱之心呵护自然生态，让吉安天更蓝、山更绿、水更清、环境更优美。

坚决打好污染防治攻坚战。抓好中央环保督察“回头看”和鄱阳湖水环境专项督察等反馈问题整改，打好八大标志性战役。推进蓝天保卫战三年行动计划，全面推行渣土密闭化运输，推动“降坡封尘”向乡镇村拓展延伸，加强秸秆禁烧与综合利用，落实《烟花爆竹燃放管理条例》，有序推进乡村禁燃禁放，确保全市PM2.5均值控制在42微克／立方米以内、空气质量优良率达到90.5%。推进水污染防治行动计划，融入长江经济带“共抓大保护”攻坚行动，落实《江西省河长制湖长制条例》，加大赣江流域生态环境综合治理和修复，完成井冈山经开区、青原区砂场混凝土搅拌区一体化建设，落实赣江河道砂石经营政府统一管理，实施峡江库区“多规合一”管控，严厉打击侵占岸线、破坏山体行为，打造赣江最美岸线。推进土壤污染防治行动计划，加强非正规垃圾堆放点整治，抓好垃圾分类试点，建成中心城区污泥处理厂，开工建设市餐厨垃圾处理中心，推动市危险废弃物处置中心、吉泰走廊生活垃圾焚烧发电厂试运行，永新、永丰、遂川、安福生活垃圾焚烧发电厂6月底前开工。

加强生态保护与修复。严守生态保护和永久基本农田保护红线，开展自然资源资产统一确权登记，加强国土空间规划管控。落实林长制，实施森林质量提升、低效林改造、湿地修复、生物多样性保护等生态工程，持续推进造林“绿化、美化、彩化、珍贵化”，加快实施国储林项目，新增人工造林20万亩、退化林修复32万亩、森林抚育100万亩。抓好自然保护区问题整改，推行采矿取土联审联批，年内基本完成被挖山体修复绿化。

完善生态文明制度体系。推进环保责任协同化，全面推行领导干部自然资源资产离任审计。推进环保工作法治化，实行环保机构垂直管理，加强生态环保综合执法，对以身试法、破坏环境的行为，以“零容忍”态度严管重罚、依法严办。推进环保主体社会化，构建政府主导、企业主体、公众参与的环境治理体系，让每个人自觉把保护环境作为一种习惯、一种责任。

（八）发展各项事业，补短板、促协调，推进社会全面进步。顺应人民对美好生活的需要，在发展中补齐社会事业短板，推进公共服务扩量提质、优质均衡。

坚持教育优先发展。落实立德树人根本任务，加强素质教育，整顿校外培训市场。实施第三期学前教育行动计划，扩大公办幼儿园、普惠性幼儿园办学规模和比例，规范城镇小区配套幼儿园建设与使用，推动中心城区城建花苑、铜锣湾中央公园、恒泰小区3个公办幼儿园秋季开学。加快义务教育优质均衡发展，改善乡村学校办学条件，基本消除城镇学校56人以上“大班额”，扩大白鹭洲中学初中部办学规模，抓好吉州区全国义务教育优质均衡发展试点。推进高中阶段教育普及，提升高等教育质量，开工建设吉安幼儿师范高等专科学校。支持民办教育发展，办好特殊教育、继续教育、国际教育，鼓励开展多层次合作办学，满足群众多样化教育需求。加强教师队伍建设，落实教师工资待遇，真正让尊师重教蔚然成风。

推进健康吉安建设。实施医疗服务体系建设三年行动，提升市直公立医院、县级综合医院、乡镇卫生院、村卫生室服务水平。发展智慧医疗，加快全民健康信息平台建设。抓好县域综合医改试点，加强医联体建设，提升家庭医生、分级诊疗服务质量，促进检查检验结果互认共享，力争县域内就诊率达90%。继续实施免费基因检测健康筛查工程。加强食品药品监管，落实‘四严’要求，建成食品药品检验检测中心，争创江西省食品安全示范城市。推动吉水、安福创建国家卫生县城，各县市区成功创建一个以上国家卫生乡镇。发展体育事业，承办体育赛事，完善体育设施，推进健身步道工程建设，促进全民健康。

加快建设文化强市。弘扬井冈山精神，推进井冈山全国理想信念教育基地建设。倡导社会主义核心价值观，深化“吉安好人”“模范市民”评选活动。健全图书馆、文化馆、美术馆、博物馆等文化设施，推动村（社区）综合性文化服务中心全覆盖，打造“书香庐陵”。加强脱贫攻坚等现实题材精品文艺创作，支持拍摄《井冈山儿女》等影视剧。繁荣哲学社会科学，发展新闻出版、广播影视、档案与地方志事业。办好“望郡吉安” 北京文物精品展，抓好永丰历史文化名城、新干大洋洲牛头城遗址保护利用，让收藏在博物馆里的文物、陈列在广袤大地上的遗产都活起来，不断扩大庐陵文化知名度。

（九）持续改善民生，兜底线、织密网，不断提升人民群众获得感幸福感安全感。牢固树立“政府过紧日子、人民过好日子”思想，严控“三公”经费，一般性财政支出压减5%以上，确保基本民生投入只增不减，促进发展成果普惠共享。

一鼓作气打好脱贫攻坚战。全市脱贫攻坚正处在滚石上山“最吃劲”的时候，必须响鼓重锤、尽锐出战。坚决抓好中央脱贫攻坚专项巡视反馈问题整改，推进脱贫攻坚三年行动，今年作为巩固提升年，要确保2.5万贫困人口脱贫、13个贫困村退出，遂川县通过考核评估，实现贫困县全部摘帽、贫困村全部退出。着力提高脱贫质量，坚持“两不愁、三保障”标准，深化精准帮扶“十二大行动”，推动农村危旧房改造全覆盖、扶贫搬迁全到位，强化“双业扶贫” “志智双扶” ，开展“感恩行动”，激发贫困群众内生动力。健全有效投入、社会保障等长效机制，摘帽不摘责任、不摘政策、不摘帮扶、不摘监管，推动井冈山市、吉安县脱贫引领地区继续走前列、作表率，永新县、万安县已脱贫摘帽地区提升脱贫成果，遂川县巩固脱贫成效。统筹推进城镇贫困群众脱贫解困，确保全面小康一个都不少。

着力促进就业。坚持就业优先，开展失业保险援企稳岗“护航行动”，落实企业吸纳残疾人就业奖补政策，促进去产能企业分流人员和受“机器换人”影响的员工转岗就业。实施高校毕业生就业促进计划和基层成长计划，积极引导农村人口进城落户。大力推进职业教育，加强终身职业技能培训，开展技能提升补贴“展翅行动”，支持社会培训机构参与政府补贴培训项目，全市新增城镇就业4.5万人、转移农村劳动力6万人。

扎牢社会保障网。深入实施全民参保计划，重点对中小微企业职工、新业态从业人员等群体实施精准扩面。稳步提高城乡居民基本医保、大病保险、城乡低保补助标准，提升特困人员供养水平，发展社会救助、社会福利、公益慈善事业。推进生育保险和职工基本医疗保险合并实施、基本医保跨省异地就医住院费用直接结算，落实抗癌药降价措施，严厉打击欺诈骗保行为。加强养老保障体系建设，推进医养结合，完善社区养老、机构养老服务，探索“社区尊老餐桌”等服务模式，让社会更温暖、更包容、更有人情味。

加强和创新社会治理。推进法治吉安建设，实施“七五”普法规划，完善公共法律服务体系；深化扫黑除恶专项斗争，严厉打击“黄赌毒”“盗抢骗”各类违法犯罪；推动基层综治中心实体化运行，创新发展“枫桥经验”，深化“访调对接”试点，促进信访工作与调解、仲裁、行政裁决、行政复议衔接配合。推进诚信吉安建设，建成个人公共信用信息数据库，着力整治政府类投资项目不及时结算、长期拖欠工程款、招商不兑现承诺等问题；健全红黑名单制度，加大失信联合惩戒力度。深化平安吉安建设，严格落实安全生产责任制，全面推行“两张表”制度，持续开展安全生产“10+1”专项整治，坚决遏制重特大安全事故发生；完善应急管理和防灾减灾救灾体制机制，切实保障人民群众生命财产安全。

扎实做好国防动员、国防教育、国家安全、人民防空等工作，推进退役军人服务体系建设，支持民主党派和工青妇、红十字会等群团组织发挥更大作用，切实做好民族宗教、外事侨务等工作。

各位代表，庐陵先贤欧阳修说过，“行之以躬，不言而信”。我们将始终坚持行胜于言、干字当头，深入推进“五型”政府建设，打造人民更加满意的政府。一是忠诚坚定勇担当。铸牢忠诚之魂，树牢“四个意识”、坚定“四个自信”，坚决做到“两个维护”，自觉把责任与担当铭刻在心，时时勿忘、久久践行。二是牢记宗旨为人民。政府干的，都应是人民盼的。要始终把人民群众对美好生活的向往作为施政方向，用脚步丈量民情、用服务增添民利，让“不为不办找理由、只为办好想办法”成为政府及其工作人员的鲜明标识。三是实干高效善作为。坚决贯彻执行中央八项规定及实施细则精神，落实“基层减负年”要求，推动“三减三强两倡导”，最大限度削减“文山会海”“材料报表”，坚决把文件压减三分之一以上，下力气解决重“痕”不重“绩”、留“迹”不留“心”问题，把干部从繁杂的事务中解脱出来。四是依法诚信塑形象。坚持职权法定，依法接受人大及其常委会的法律监督和工作监督，自觉接受人民政协的民主监督，主动接受监察、司法和舆论等各方面的监督，认真听取民主党派、工商联、无党派人士和各人民团体的意见，言必信、行必果。五是清正廉明当表率。一体推进不敢腐、不能腐、不想腐，严惩公共资源交易、国资国企改革、脱贫攻坚、金融等领域腐败行为，严查发生在群众身边的不正之风和“微腐败”，对踩“红线”、越“底线”、闯“雷区”的一切违规违纪违法行为，不以权势大而回避，不以问题小而姑息，不以违者众而放任，不断巩固和发展反腐败斗争的压倒性胜利。

各位代表！新时代的浪潮滚滚向前，惟拼搏者强，惟奋斗者赢；金庐陵的美好画卷徐徐铺展，惟奔跑者进，惟实干者成。让我们更加紧密地团结在以习近平同志为核心的党中央周围，在省委、省政府和市委的坚强领导下，以更加昂扬的斗志、更加饱满的热情、更加旺盛的干劲，奋力推进吉安高质量跨越式发展，加快实现“三个走在前列、两大战略任务”，以优异成绩庆祝新中国成立70周年!
